# Effect of a Novel Photoelectrochemical Oxidation Air Purifier on Nasal and Ocular Allergy Symptoms

**DOI:** 10.1177/2152656718781609

**Published:** 2018-06-21

**Authors:** Nikhil G. Rao, Ambuj Kumar, Jenny S. Wong, Ravi Shridhar, Dharendra Y. Goswami

**Affiliations:** 1 Molekule, Inc., San Francisco, California; 2 University of South Florida College of Medicine, Tampa, Florida; 3 University of Central Florida College of Medicine, Orlando, Florida; 4 Department of Chemical and Biomedical Engineering, University of South Florida, Tampa, Florida

**Keywords:** allergy, asthma, air purifier, portable, photoelectrochemical oxidation, portable, nasal, ocular, sleep

## Abstract

**Background:**

Photoelectrochemical oxidation (PECO) is a new air purification technology developed to reduce circulating indoor allergens. PECO removes particles as small as 0.1 nm with the destruction of organic matter otherwise not trapped by a traditional filter and removes volatile organic compounds.

**Objective:**

We hypothesized that with daily use, the device would reduce user nasal and ocular allergy total symptom scores (TSS) within 4 weeks.

**Methods:**

The study was performed among 46 individuals with self-reported allergies using a portable PECO air purifier. Self-reported TSS were calculated at baseline and weekly for 4 weeks following initiation of continuous use of the system. TSS was the sum of total nasal symptom scores (TNSS) and total ocular symptom scores (TOSS) for the week.

**Results:**

There was a statistically significant change in overall TSS from baseline to 4 weeks (10.1 at baseline and 4.35 postintervention) resulting in a mean difference of 5.75 (95% confidence interval [CI] 4.32–7.18; *P* < .0001). There was a statistically significant change in TNSS from baseline to 4 weeks (6.3 at baseline and 3.04 postintervention) resulting in a mean difference of 3.26 (95% CI 2.33–3.19; *P* < .0001). There was a statistically significant change in TOSS from baseline to 4 weeks (3.82 at baseline and 1.3 postintervention) resulting in a mean difference of 2.52 (95% CI 1.74–3.3; *P* < .0001).

**Conclusion:**

With the use of PECO air purification technology, TSS, TNSS, and TOSS decreased significantly. These improvements were consistent over the 4-week course of device use.

## Introduction

In the United States, the incidence of respiratory allergies and asthma is increasing, with 10% to 40% of the population suffering from allergies^1^ and 8% suffering from asthma.^2^ The direct and indirect health costs and decrement in quality of life from these illnesses are substantial.^2,3^ While these symptoms are typically attributed to aeroallergens, there are particulate matter and volatile organic compounds (VOCs) that act as irritants that can also evoke symptoms. Local air filtration has shown some ability to decrease allergen counts in the air and thus improve the symptoms experienced by allergy and asthma sufferers under certain conditions^4^. However, to date, the efficacy of a comprehensive air purification system, particularly with high-efficiency particulate air (HEPA) filtration, as a sole intervention modality has been equivocal and the extent of air filtration remains suboptimal.

Photoelectrochemical oxidation (PECO) is a revolutionary new technology for providing an air purification solution. In addition to physical filtration, a photoelectrochemical reaction takes place on the surface of a nano-coated filter leading to the oxidation of organic matter. These processes allow for the destruction of organic material 1000 times smaller than what a HEPA filter can capture.^5,6^ Thus, PECO not only removes but can also efficiently destroy organic matter, bacteria, viruses, mold, and VOCs converting them into their trace elements.^7^

In this report, we present the results of our initial experience using portable PECO air purification technology in the home with users who complained of respiratory allergy symptoms and some of whom also suffered from asthma.

## Description of PECO Technology (molekule.com)

PECO is a catalytic oxidation reaction in which photons of light that have energy more than the bandgap of a photocatalyst excite the photocatalyst which produces hydroxyl free radicals in the presence of water molecules in air. The hydroxyl free radicals are extremely potent oxidizers, which oxidize organics and microorganisms in air to form CO_2_ and water and trace minerals. Equations (1) to (5) describe the chemical reactions.

(1) $$Photocatalyst+\text{Photon}(h\nu )\to hole_{vb}^{+}+e^{-}$$

(2) $${\text{H}}_{\text{2}}\text{O}\to {\text{OH}}^{-}+{\text{H}}^{\text{+}}$$

(3) $${\text{hole}}_{\text{vb}}^{+}+{\text{OH}}_{\text{ads}}^{-}\to {\text{OH}}^{•}$$

(4) $$2{\text{H}}^{+\;}2{\text{e}}^{-}+\;\frac{1}{2}{\text{O}}_{2}\;\to \;{\text{H}}_{2}\text{O}$$

Oxidation of organics

(5) $${\text{OH}}^{•}+\text{Organics}+{\text{O}}_{2}\to \text{Products}\left({\text{CO}}_{2},{\text{H}}_{2}\text{O},\text{etc}.\right)$$

The pioneering work in the field of photocatalytic disinfection of indoor air was done by Goswami et al. when they developed a technology to completely destroy biological contaminants in indoor air.^8,9^ Wolfrum et al. demonstrated complete mineralization of *Escherichia coli*, *Micrococcus luteus*, *Bacillus cereus* (bacterial cells and spores), and *Aspergillus niger* spores by photocatalytic oxidation.^10^ They based their results on kinetic data and carbon mass balance. Goswami later enhanced the process by separating the electrons and holes by photoelectrochemical process that improved the effectiveness of photocatalytic oxidation by orders of magnitude, which is the underlying technology of Molekule device. Goswami and his coworkers published a total of 18 peer-reviewed papers in scientific journals. In a final paper, Goswami and his group explained the whole disinfection process by PECO.^11^

## Methods

We performed a prospective cohort study evaluating the use of a portable air purifier with PECO technology from March 2015 to April 2017. The study was approved by the institutional review board at IntegReview, Austin, TX and written consent was obtained. Consecutive adult subjects older than 18 volunteered to test the unit for a 1-month trial period. Volunteers were not paid and were identified through social media outreach. All subjects expressed interest in testing the new air purification technology to see if it helped their allergies and/or asthma.

All subjects had some degree of nasal or ocular allergy symptoms and some also suffered from asthma symptoms. Some participants primarily agreed to test the unit to see if it helped with their sleep or overall quality life. Instructions were given that participants should use the air purifier for a minimum of 12 h/day and preferably at nighttime with the unit close to the bed if possible. During the study duration, participants were advised to continue their normal medications for allergic symptoms, asthma, and any other general medical condition and to continue their usual routine for managing allergies and asthma. The duration of the study was 4 weeks. Symptoms were self-recorded weekly.

### Outcome Measures

The primary outcome for the study was change in overall symptom scores from baseline to the scores at 4 weeks. The secondary outcomes were change in overall symptom score, total nasal symptom scores (TNSS), total ocular symptom scores (TOSS), and sleep quality from baseline to the 1- and 4-week time points, and change in asthma symptoms from baseline to the 4-week time point. Data on all outcomes were collected at baseline and weekly over 4 weeks via a web-based survey tool. The TNSS and TOSS tool is a validated tool and has been widely used. Briefly, TNSS consist of patient rating of the degree of nasal congestion, runny nose, nasal itchiness, and sneezing, while TOSS consist of patient rating of the degree of eye itchiness, eye wateriness, and eye redness.^12,13^ Both are graded on a scale of 0 to 3 where 0 represented no symptoms, 1—mild symptoms, 2—moderate symptoms, and 3—severe symptoms. Total symptom scores (TSS) are the sum of TNSS and TOSS. Participants with at least some moderate nasal or ocular symptoms and TSS of 8 or greater at baseline were considered to have active respiratory allergies (allergy subjects). Sleep quality was assessed on a scale of 0 to 3. In the past 4 weeks, please rate how difficult sleep has been with nasal symptoms on a scale from 0 to 3 (0—none, 1—mild, 2—moderate, and 3—severe). Asthma symptoms were recorded on a 0- (minimum) to 4- (maximum) point scale at baseline and at 4 weeks. A point was given for poor control for each question over the past 4 weeks. Questions assessed missed work or daily activities due to asthma, waking up, whether asthma was felt to be well controlled, and greater than 12 puffs inhaler use per day.^14^

Self-reported medication use for allergies and asthma, dose, frequency, and route were assessed at baseline and at 4 weeks.

### Statistical Analysis

Descriptive statistics (eg, frequency and relative percentages, means, and standard deviations [SDs]) were used to describe demographic characteristics of included subjects. The change in outcomes following intervention was compared using paired *t* tests and summarized as mean differences along with 95% confidence intervals (CIs). For the ease of interpretation, summary measures from continuous data were converted into odds ratio along with 95% CI.^15^ The statistical significance was set at *P* < .05 for all comparisons. All analyses were performed using SPSS statistical analysis software version 23.

## Results

### Participant Characteristics

A total of 49 adult patients volunteered to participate in using the portable air purifier. Forty-seven percent of the participants were male (n = 23) and 53% were females (n = 26) (Table 1). The mean age of the participants was 39.8 years (SD ± 12.6; range 18–77 years). The majority of the participants (73%; n = 36) had active allergies with baseline TSS greater than or equal to 8. Some of the participants had, in addition to allergies, a history of asthma (36%; n = 18).

**Table 1.** Participants Characteristics.

Variables	N (%)
Gender	
Female	27 (55.1)
Male	22 (44.9)
Age	
Mean (range)	40 (18–77)
Race	
Asian or Pacific Islander	6 (12.2)
Black of African American	1 (2.0)
Hispanic or Latino	5 (10.2)
White/Caucasian	36 (73.5)
Middle Eastern	1 (2.0)
Active allergy symptoms	
Yes	38 (77.5)
No	11 (22.5)
Asthma	
Yes	18 (36.7)
No	31 (63.2)
Medication allergies and asthma	
Yes	37 (75.5)
No	12 (24.5)

### Outcomes

All subjects were compliant with using the air purifier for 1 month as planned. Forty-six of the 49 submitted fully completed survey questions at baseline and at 4 weeks for analysis.

Of the 49 participating subjects, 46 subjects completed the trial, defined as having recorded the data on all outcomes at 4 weeks postintervention. Eighty-nine percent of asthma sufferers had data on all outcomes at 4 weeks (n = 16). For week 1 assessments, data were available on all 49 subjects.

### Overall Symptom Score Results at Weeks 4 and 1

**Figure 1.**
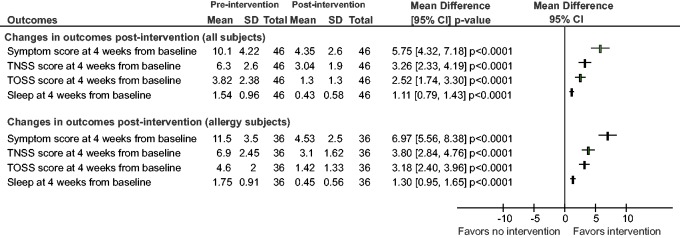
Forest plot showing changes in outcomes at 4 weeks from baseline postintervention. Statistical improvements were seen in total symptoms scores (TNSS + TOSS), total nasal symptom scores (TNSS), total ocular symptom scores (TOSS), and sleep scores. Greater improvements were seen in subjects with active respiratory allergies.

**Figure 2.**
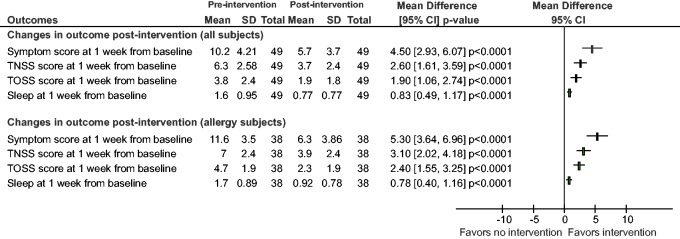
Forest plot showing changes in outcomes at 1 week from baseline postintervention. Statistical improvements were seen in total symptoms scores (TNSS + TOSS), total nasal symptom scores (TNSS), total ocular symptom scores (TOSS), and sleep scores. Greater improvements were seen in subjects with active respiratory allergies.

As indicated in Figure 1, there was a statistically significant change in overall TSS (TNSS + TOSS) from baseline to the 4-week time point (10.1 at baseline and 4.35 postintervention) resulting in a mean difference of 5.75 (95% CI, 4.32–7.18; *P* < .0001). The resultant odds ratio was 19.5 (95% CI, 8.09–44.6) Indeed, all symptom elements within TNSS and TOSS showed improvement (nasal congestion, itchiness, sneezing, runny nose, eye redness, secretion, and itchiness) (*P* < .001). Among subjects with active respiratory allergies (n = 36), there was a statistically significant change in overall symptom score from baseline to the 4-week time point (11.5 at baseline and 4.53 postintervention) resulting in a mean difference of 6.97 (95% CI, 5.56–8.38; *P* < .0001). The resultant odds ratio was 58.6 (95% CI, 19.3–162.5). As indicated in Figure 2, there was a statistically significant change in overall symptom score from baseline to the 1-week time point (10.2 at baseline and 5.7 postintervention) resulting in a mean difference of 4.5 (95% CI, 2.93–6.07; *P* < .0001). The resultant odds ratio was 7.8 (95% CI, 3.55–16.6). Again, all symptom elements showed improvement (nasal congestion, itchiness, sneezing, runny nose, eye redness, secretion, and itchiness) (*P* < .001). Among subjects with active allergies (n = 38), there was a statistically significant change in overall symptom score from baseline to the 1-week time point (11.6 at baseline and 6.3 postintervention) resulting in a mean difference of 5.3 (95% CI, 3.64–6.96; *P* < .0001). The resultant odds ratio was 13.5 (95% CI, 5.3–32.7). Improvements seen at 1 week continued for the entire 4-week testing period. Forty-three subjects had improved and 3 subjects had worse TSS (baseline to exit, 1–8, 4–5, and 3–8). These changes were not statistically significant. Subjects with allergies and allergies/asthma both had reductions in TSS that were statistically significant (*P* < .005). At 4 weeks, in the allergy group, there was a mean change in score of 6.1 (initial score 10.4) and in the allergy/asthma group, there was a mean change in score of 5.2 (initial score 9.9).

### TNSS Results at Weeks 4 and 1

There was a statistically significant change in TNSS from baseline to the 4-week time point (6.3 at baseline and 3.04 postintervention) resulting in a mean difference of 3.26 (95% CI, 2.33–3.19; *P* < .0001; see Figure 1). The resultant odds ratio was 13.3 (95% CI, 5.7–29.9). Among subjects with active allergies (n = 36), there was a statistically significant change in TNSS from baseline to 4 weeks (6.9 at baseline and 3.1 postintervention) resulting in a mean difference of 3.8 (95% CI, 2.84–4.76; *P* < .0001). The resultant odds ratio was 27.4 (95% CI, 9.8–71.4). There was also a statistically significant change in TNSS from baseline to the 1-week time point (6.3 at baseline and 3.7 postintervention) resulting in a mean difference of 2.6 (95% CI, 1.61–3.59; *P* < .0001; see Figure 2). The resultant odds ratio was 6.61 (95% CI, 3.03–13.97). Among subjects with active allergies (n = 38), there was a statistically significant change in TNSS from baseline to the 1-week time point (7 at baseline and 3.9 postintervention) resulting in a mean difference of 3.1 (95% CI, 2.02–4.18; *P* < .0001). The resultant odds ratio was 10.35 (95% CI, 4.14–24.71).

### TOSS Results at Weeks 4 and 1

There was a statistically significant change in TOSS from baseline to the 4-week time point (3.82 at baseline and 1.3 postintervention) resulting in a mean difference of 2.52 (95% CI, 1.74–3.3; *P* < .0001; see Figure 1). The resultant odds ratio was 10.8 (95% CI, 4.7–23.9). Among subjects with active allergies (n = 36), there was a statistically significant change in TOSS from baseline to the 4-week time point (4.6 at baseline and 1.42 postintervention) resulting in a mean difference of 3.18 (95% CI, 2.4–3.96; *P* < .0001). The resultant odds ratio was 29.6 (95% CI, 10.5–77.6*).* There was also a statistically significant change in TOSS from baseline to the 1-week time point (3.80 at baseline and 1.90 postintervention) resulting in a mean difference of 1.90 (95% CI, 1.06–2.74; *P* < .0001; see Figure 2). The resultant odds ratio was 5.05 (95% CI, 2.35–10.59). Among subjects with active allergies (n = 38), there was a statistically significant change in TOSS from baseline to 1 week (4.7 at baseline and 2.3 postintervention) resulting in a mean difference of 2.4 (95% CI, 1.55–3.25; *P* < .0001). The resultant odds ratio was 9.83 (95% CI, 3.94–2.4).

### Sleep Quality at Weeks 4 and 1

There was a statistically significant change in sleep quality from baseline to the 4-week time point (1.54 at baseline and 0.43 postintervention) resulting in a mean difference of 1.11 (95% CI, 0.79–1.43; *P* < .0001; see Figure 1). The resultant odds ratio was 12.1 (95% CI, 5.2–27.9). Among subjects with active allergies (n = 36), there was a statistically significant change in sleep quality from baseline to the 4-week time point (1.75 at baseline and 0.45 postintervention) resulting in a mean difference of 1.3 (95% CI, 0.95–1.65; *P* < .0001). The resultant odds ratio was 22.5 (95% CI, 8.2–57.8). There was a statistically significant change in sleep quality from baseline to the 1-week time point (1.6 at baseline and 0.77 postintervention) resulting in a mean difference of 0.83 (95% CI, 0.49–1.17; *P* < .0001; see Figure 2). The resultant odds ratio was 5.7 (95% CI, 2.63–11.9). Among subjects with active allergies (n = 38), there was a statistically significant change in sleep quality from baseline to the 1-week time point (1.7 at baseline and 0.92 postintervention) resulting in a mean difference of 0.78 (95% CI, 0.4–1.16; *P* < .0001). The resultant odds ratio was 5.40 (95% CI, 2.26–12.05).

### Asthma Control (n = 16) at 4 Weeks

There was a statistically significant change in asthma symptoms from baseline to those seen at 4 weeks (2.06 at baseline and 0.75 postintervention) resulting in a mean difference of 1.31(95% CI, 0.45–2.18; *P* = .006).

#### Medication Use and Adverse Events

Allergy and asthma medication use at baseline was reported in Table 1. At 4 weeks, 34 of the 37 participants who were taking medications provided medication use details; 67.7% (n = 23) reported a decrease in medication use and 32.3% (n = 11) reported no decrease in medication use (*P* = .006). Adverse events related to the air purifier were not reported. Two subjects complained of headaches, 1 due to a sinus infection and 1 who had preexisting headaches at baseline. Six complained of light and 14 complained of noise.

## Discussion

In our study, we evaluated the clinical efficacy of using a portable home air purifier with a novel air filtration technology, PECO. Our results demonstrate a significant improvement in nasal-related allergy symptoms and ocular-related allergy symptoms in those who used it daily. The significant improvements were seen after 1 week of use of the air purifier. Moreover, the symptom reductions that were seen were sustained with continuous use at 4 weeks after initiation. All symptom elements showed significant improvement during weeks 1 to 4 (nasal congestion, itchiness, sneezing, runny nose, eye redness, secretion, and itchiness). In addition, we noted significant improvements in sleep quality after use of the air purifier at weeks 1 to 4. Total symptom reduction and improvements in sleep were even more profound in those with at least moderate, active allergy symptoms at baseline. A small subset of individuals who tried the air purifier also had a history of asthma. For those who reported asthma symptoms, there was a significant improvement in their symptoms after 4 weeks of use.

**Figure 3.**
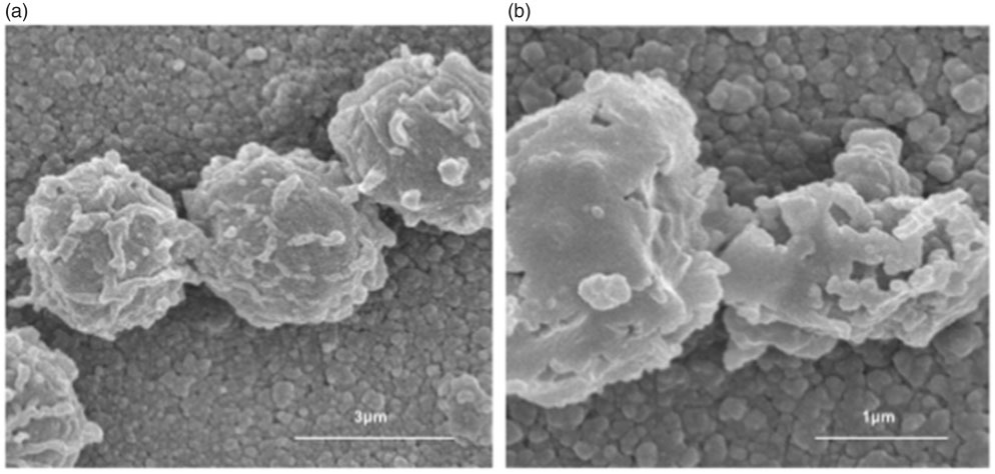
*Aspergillus niger* spores (A) In dark (B) being oxidized with PECO as noted on electron microscopy.^9^

**Figure 4.**
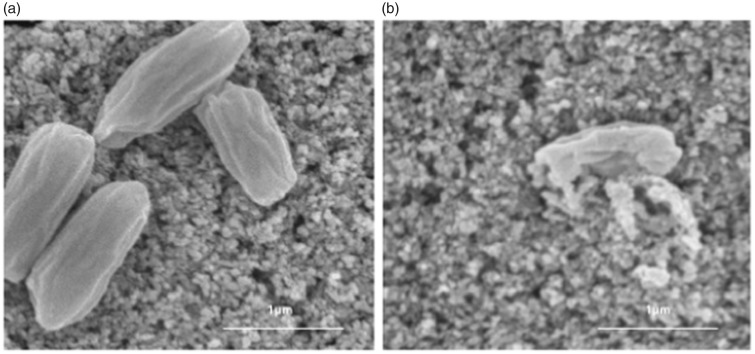
*Bacillus subtilis* endospores (A) In dark (B) being oxidized with PECO as noted on electron microscopy.^9^

PECO is an air purification technology that destroys pollutants 1000 times smaller than HEPA filters can trap.^5,6^ The technology works by emitting ultraviolet-A light on to a filter membrane coated with nanoparticles. This creates a photoelectrochemical reaction on the surface of the filter that will break down the molecular structure of organic particles in the air. Although a traditional filter can only collect pollutants on the filter surface such that they can potentially reenter the air stream, PECO destroys pollutants as small as 0.1 nm. HEPA filters by contrast can only capture pollutants efficiently down to 300 nm in size.^16^ We felt that given the exponential improvement in PECO technology’s filtering ability that there could be clinical benefits in symptoms related to allergy, VOCs, and particulate matter exposure in the home, and this would amount to a preventative strategy that could not only improve outcomes but decrease over-the-counter and prescription medication usage, since these triggers are effectively reduced. Prior to the current study, we have shown in detail the capacity of this filtration technology but have not pursued studies of clinical efficacy (see Figures 3 and 4).^5,7^

Despite improvements in therapy and drug delivery for patients with respiratory allergies and asthma, there is a significant need for improvement in the overall treatment strategy that may also include preventive methods such as air filtration. For example, up to 50% of asthmatics are not under optimal control.^17,18^ Seasonal or perennial allergic symptoms are present in 10% to 40% of the U.S. population, resulting in at least 6 billion dollars in overall health-care expenditures per year ^1,19^. In addition, compliance with medication use can be problematic and for some classes of drugs, there may be long-term adverse events.^20^ For these reasons, various environmental interventions have been evaluated to further address these problems.

One of the landmark studies for asthma patients using multiple environmental interventions was the inner-city asthma study group randomized trial.^21^ This study included the use of HEPA air filtration in the child’s bedroom if exposed to secondhand smoke or sensitized to cat, dog, or mold allergens. It appeared that comprehensive intervention which included air filtration as well as allergen covers, vacuum cleaning, and pest control helped reduce asthma-related exacerbations and symptoms, but the specific improvements that may have been attributable to air purification alone were not evaluated.

Several additional studies have evaluated the role of air filtration alone in improving respiratory allergy symptoms and asthma. A meta-analysis analyzing the 10 trials that were performed between 1973 through 1999 including asthma patients reported significantly lower TSS and lower sleep disturbance score; however, heterogeneity of results weakened the inferences from these trials.^22^ Some trials have specifically looked at the clinical benefit of using portable room air purifiers as compared to whole home filtration. In this context, while air purification with HEPA filtration has provided a variable degree of benefit for some individuals, ionic electrostatic room air cleaners appear to be of no benefit and may produce ozone, a potentially harmful respiratory irritant.^4^ In a randomized trial using portable HEPA air cleaners and HEPA vacuum cleaning in the bedroom and living room in 30 asthmatics living with an indoor cat or dog, there were statistical improvements in asthma outcomes including bronchial reactivity and treatment requirements.^23^ In another study by Gore et al., HEPA portable units reduced the amount of cat allergen in the room. However, this effect was mitigated when the cat was removed from the room.^24^ Batterman et al. reported a 2-month long study evaluating the effects of HEPA portable units in the homes of cigarette smokers. Results showed a reduction in particulate matter concentrations; however, clinical effects were not studied.^25^ Sulser et al. evaluated children sensitized to cat or dog allergens who used portable air purification with HEPA technology in the living room and bedroom in a randomized controlled trial.^26^ Although HEPA air cleaners retained airborne pet allergens, no effect on disease activity or allergen concentrations in bulk dust samples was observed. Randomized studies have more recently looked at the use of HEPA filtration in the breathing zone utilized during sleep. Pedroletti et al. evaluated the use of HEPA filtration in the sleep breathing zone for teenagers and young adults concluding that clean air, administered directly to the breathing zone during sleep, can have a positive effect on bronchial inflammation and quality of life.^27^ Stillerman et al. utilized a combination of HEPA filtration along with a dust mite proof pillowcase. Significant improvements were seen in nocturnal nasal and ocular allergy symptoms and quality of life for the active versus placebo device.^16^

While there generally appears to be some improvement in disease management for both allergy and asthma sufferers using HEPA filtration, there remain several notable drawbacks to this technology. Even with maximum filtering efficiency, particulate matter cannot be completely filtered from the air by HEPA.^28^ In addition, it is unclear to what extent HEPA filtration can decrease mold spore counts, if at all, and may allow for these spores to recirculate in the air exacerbating allergy and asthma.^29,30^ A major drawback of HEPA filtration is that it is not able to remove the smaller allergens bacteria, viruses, and VOCs that are smaller than 300 nm.^16^ This group can be the source of allergies, infection, and respiratory irritation leading to allergy or asthma exacerbation, which have yet to be included in filtration methods to date for the prevention or improvement of these symptoms. In contrast, PECO technology allows for both physical filtration, like with HEPA, and photocatalysis to oxidize organic material into its trace elements, notably water and carbon dioxide. Unlike HEPA filtration, PECO technology has been shown to completely oxidize and destroy pollutants such as mold, bacteria, viruses, and VOCs and thus represents substantial improvement in air filtration over any technology available in the home.^5,7^

Our current study has several strengths and limitations. The main limitation is the lack of a comparator and being a pre–post study, the findings are subject to regression to the mean. To conclusively address the efficacy of the air purifier, an adequately powered, designed, and executed randomized controlled trial is needed. Although we did not have a “placebo” arm, each person does serve as their own control, which is not unlike other studies in the field.^31^ We were unable to differentiate allergic versus nonallergic rhinitis or asthma subjects due to the limitations of our current study design. We also knew upfront that we would be unable to measure levels of allergens and fully assess or control other environmental interventions used by the subjects given their heterogeneity. This issue can be conclusively addressed only in a randomized controlled trial where groups would be balanced for the environmental factors. We utilized self-reporting of symptoms, which is not unusual for allergy studies. However, for asthma, there are objective measurements that can now be performed at home such as forced expiratory volume and peak expiratory airflow. Future studies could incorporate such measurements, especially with a larger group of asthmatics. In such a study, it may be prudent to phenotype patients prior to study so as to understand which of the various asthmatic subgroups reap the greatest benefit. Such a study is beyond the scope of the current work. Nevertheless, asthmatics in our study showed fewer asthma-related symptoms, strongly indicative of a positive effect. We contend that an even greater magnitude of symptom score reduction would have been seen with subjects who have worse allergic symptoms, but this awaits explicit testing.

In conclusion, we found significant and sustained improvements in respiratory allergy symptoms within a week of using portable air filtration using PECO technology. Improvements were also noted in sleep quality. There was a benefit after 1-week use which was sustained for the entire 4-week use of the air purifier. In the subset of those suffering from asthma, there appeared to be an improvement in asthma-related symptoms. In summary, PECO is a novel technology that could be very useful in the future management of respiratory allergies and asthma.

## Authors’ Note

This work has been presented as a poster at the American College of Allergy, Asthma, and Immunology Scientific Meeting, Boston, MA on October 27, 2017.
